# First Complete Genome Sequences of Zika Virus Isolated from Febrile Patient Sera in Ecuador

**DOI:** 10.1128/genomeA.01673-16

**Published:** 2017-02-23

**Authors:** S. Márquez, J. Carrera, S. T. Pullan, K. Lewandowski, V. Paz, N. Loman, J. Quick, D. Bonsall, R. Powell, J. Thézé, O. G. Pybus, P. Klenerman, J. Eisenberg, J. Coloma, M. W. Carroll, G. Trueba, C. H. Logue

**Affiliations:** a Colegio de Ciencias Biológicas y Ambientales, Universidad San Francisco de Quito, Quito, Ecuador; b Public Health England, Porton Down, United Kingdom; c Clinical Laboratory, Hospital Delfina Torres de Concha, Esmeraldas, Ecuador; d Institute of Microbiology and Infection, University of Birmingham, Birmingham, United Kingdom; e Peter Medawar Building for Pathogen Research, University of Oxford, Oxford, United Kingdom; f Primer Design Ltd., Southampton, United Kingdom; g Department of Zoology, University of Oxford, Oxford, United Kingdom; h School of Public Health, University of Michigan, Ann Arbor, Michigan, USA; i University of California, Berkeley, Berkeley, California, USA

## Abstract

Here, we present the complete genome sequences of two Zika virus (ZIKV) strains, EcEs062_16 and EcEs089_16, isolated from the sera of febrile patients in Esmeraldas City, in the northern coastal province of Esmeraldas, Ecuador, in April 2016. These are the first complete ZIKV genomes to be reported from Ecuador.

## GENOME ANNOUNCEMENT

The emergence and diagnostic detection of arthropod-borne viruses have been increasingly reported in Ecuador, and include dengue virus (DENV), chikungunya virus (CHIKV), and, most recently, Zika virus (ZIKV) (1). In 2016 alone, 2,693 suspected, 839 laboratory-confirmed, and 15 imported cases of ZIKV were reported (2). The early clinical manifestations resulting from infection with ZIKV closely resemble those caused by DENV and CHIKV (3), and without differential diagnosis by serological, molecular, or sequencing means, misdiagnosis based on clinical presentation alone may occur (4, 5).

Here, we report the complete genome sequences of two ZIKV strains, EcEs062_16 and EcEs089_16, isolated from the sera of two febrile patients in the coastal province of Esmeraldas, Ecuador, in April 2016. ZIKV was detected in both patient sera samples using the Genesig Dengue, Zika and Chikungunya Virus Multiplex real-time assay kit on a BioRad CFX96 system, following the manufacturer’s guidelines (Genesig, United Kingdom).

ZIKV was propagated from sera by inoculation of C6/36 cell monolayers (*Aedes albopictus*; ECACC, United Kingdom). Supernatant was removed after 7 days, purified, and nucleic acid extracted using the QIAamp viral RNA minikit (Qiagen Gmbl, Germany); 5 μl of purified RNA was used as template in ten 20-μL reactions, each with a set of 10 primer pairs designed to amplify ~1.5-kb overlapping amplicons covering the whole genome. Sample EcEs089_16 produced insufficient product from the 1.5-kb amplicon scheme, and therefore the 400-bp tiling amplicon protocol devised by Quick and Loman (http://www.zibraproject.org/data) was utilized to produce sufficient product for sequencing. Sequencing libraries were prepared from 1 μg of total material, comprising equimolar amounts of each of the 10 amplicons for EcEs062_16 or 500 ng of product from each of the two multiplex reactions for EcEs089_16.

The Nanopore sequencing kit SQK-NSK007 (Oxford Nanopore Technologies, United Kingdom) was used to produce both sequencing libraries, according to the manufacturer’s R9 amplicon sequencing protocol. EcEs062_16 and EcEs089_16 libraries were sequenced on FLO_MIN104 and FLO_MIN105 flow cells, respectively. Sequencing was performed on an Mk1b MinION device. Bases were called in Metrichor using 2D Basecalling RNN for SQK-NSK007. Consensus sequences of 10,646 bp and 10,616 bp for EcEs062_16 and EcEs089_16, respectively, were generated using the ZiBRA analysis pipeline (http://www.zibraproject.org/data). Direct RNA sequencing of culture supernatant using Illumina technology provided fuller length sequences of 10,812 bp and 10,810 bp, respectively, for EcEs062_16 and EcEs089_16.

The EcEs062_16 and EcEs089_16 genomes clustered together with a sequence isolated from Paraiba state, Brazil (KX280026, posterior probability 0.96, bootstrap support 75%), rather than with sequences from neighboring Colombia and Peru. Esmeraldas city is one of the furthest continental points west of the Paraiba region (4,874 km by air). Neither patient reported travel history to Brazil, and Ecuador does not share a land border with Brazil; therefore, the epidemiology of ZIKV’s introduction into and movement within Ecuador merits further investigation. We aim to further validate the potential of portable sequencing as a diagnostic tool to assess the temporal movements of ZIKV throughout South America, as we previously described for Ebola in West Africa (6).

### Accession number(s).

The complete genomic sequences have been annotated and deposited in GenBank, under accession numbers KX879603 to KX879604, and on http://nextstrain.org/zika.
